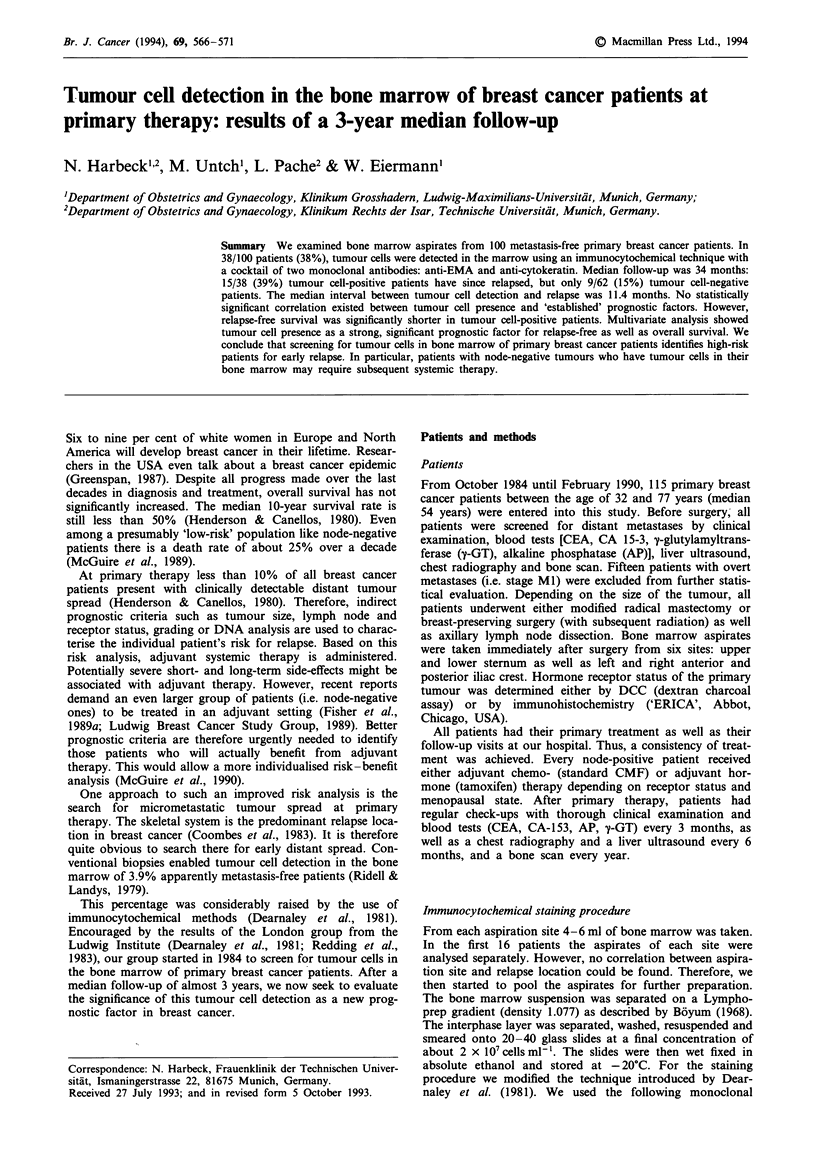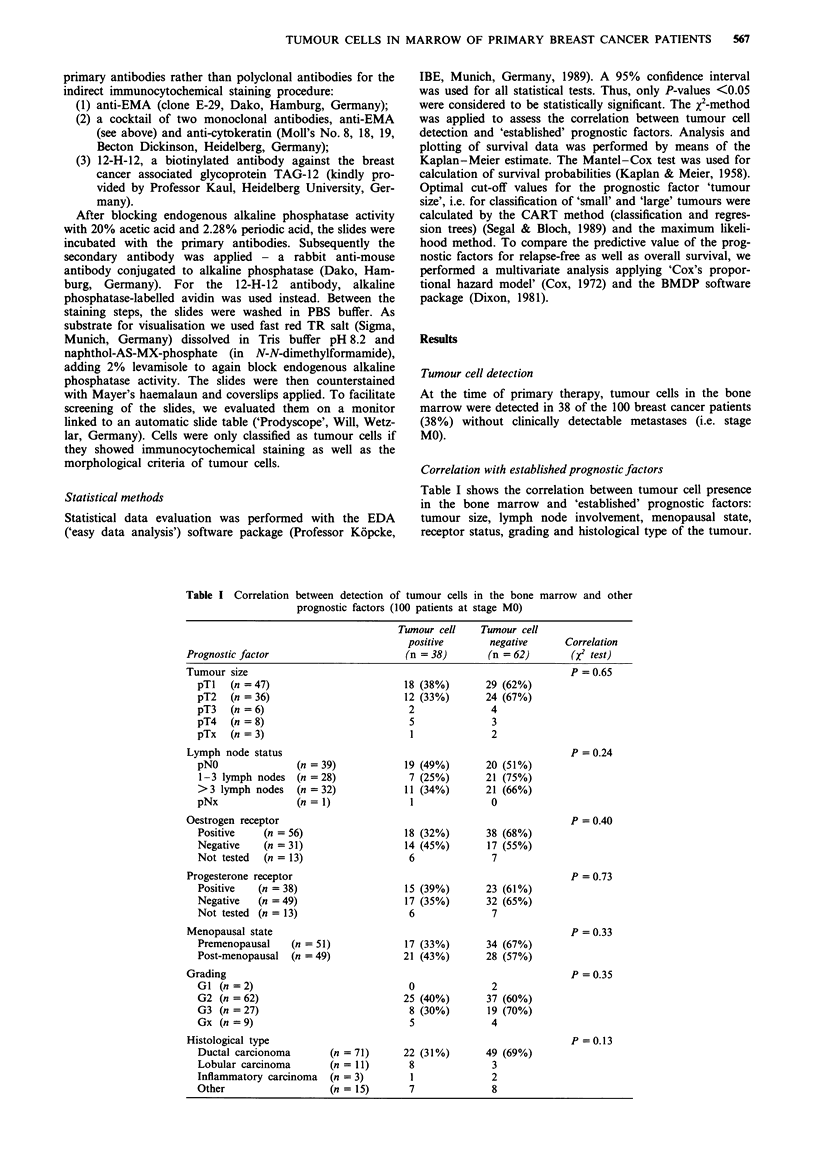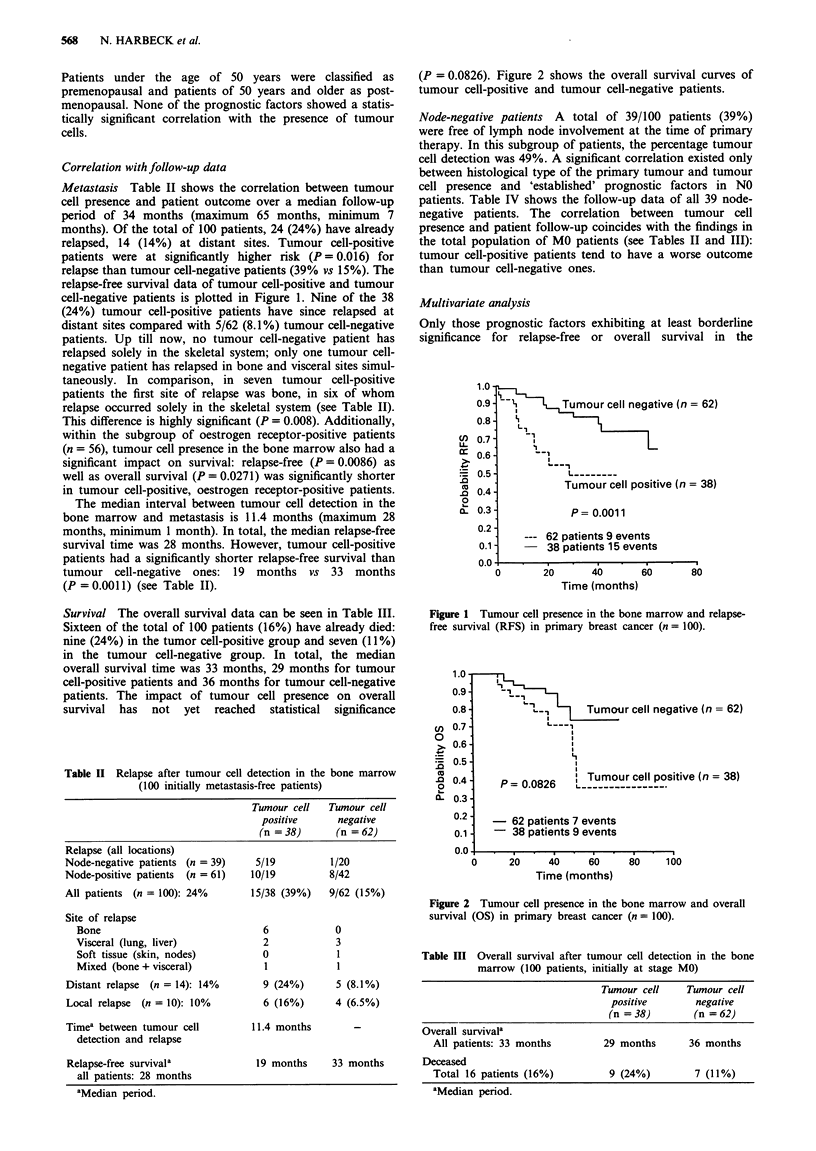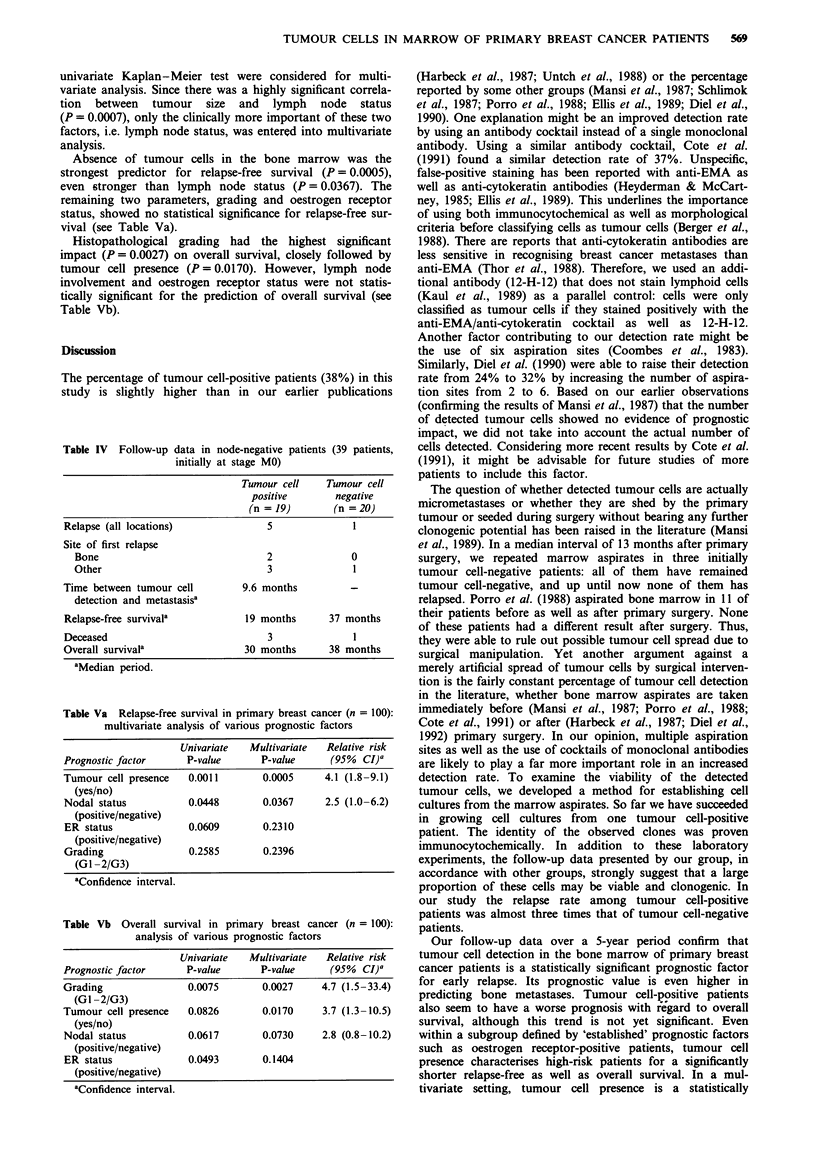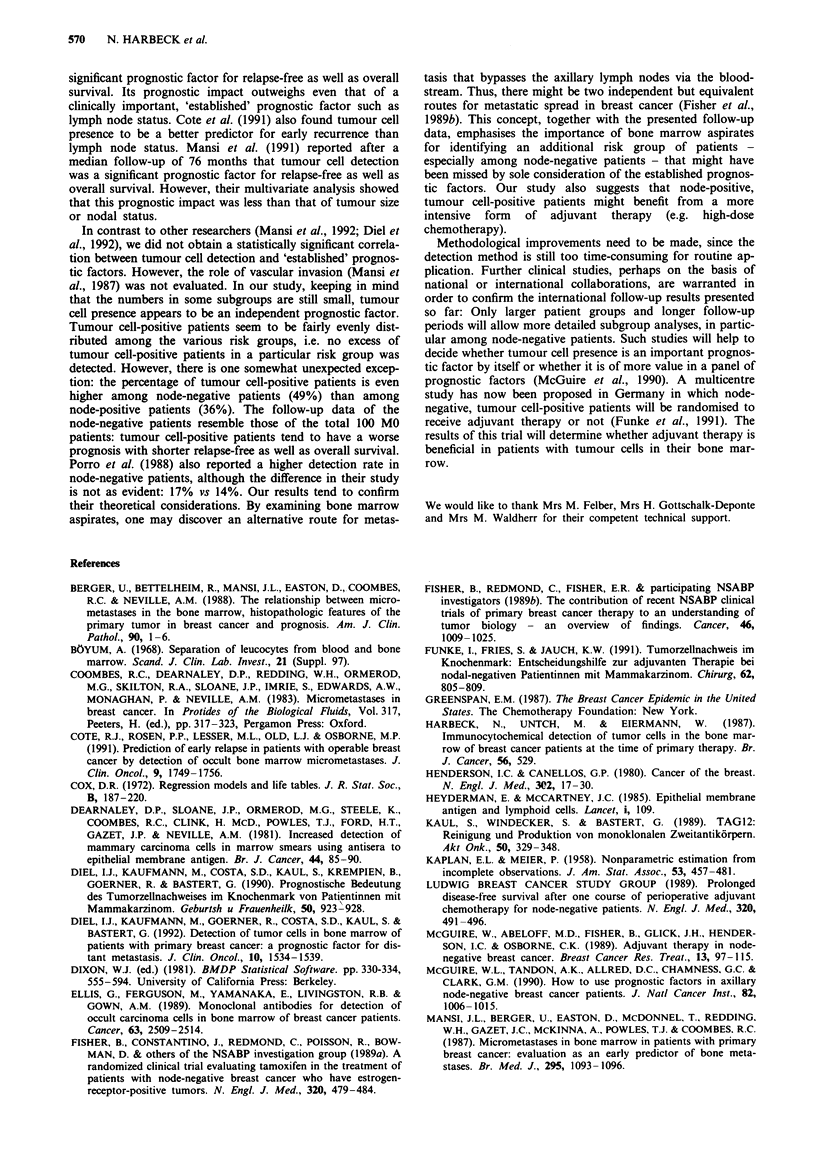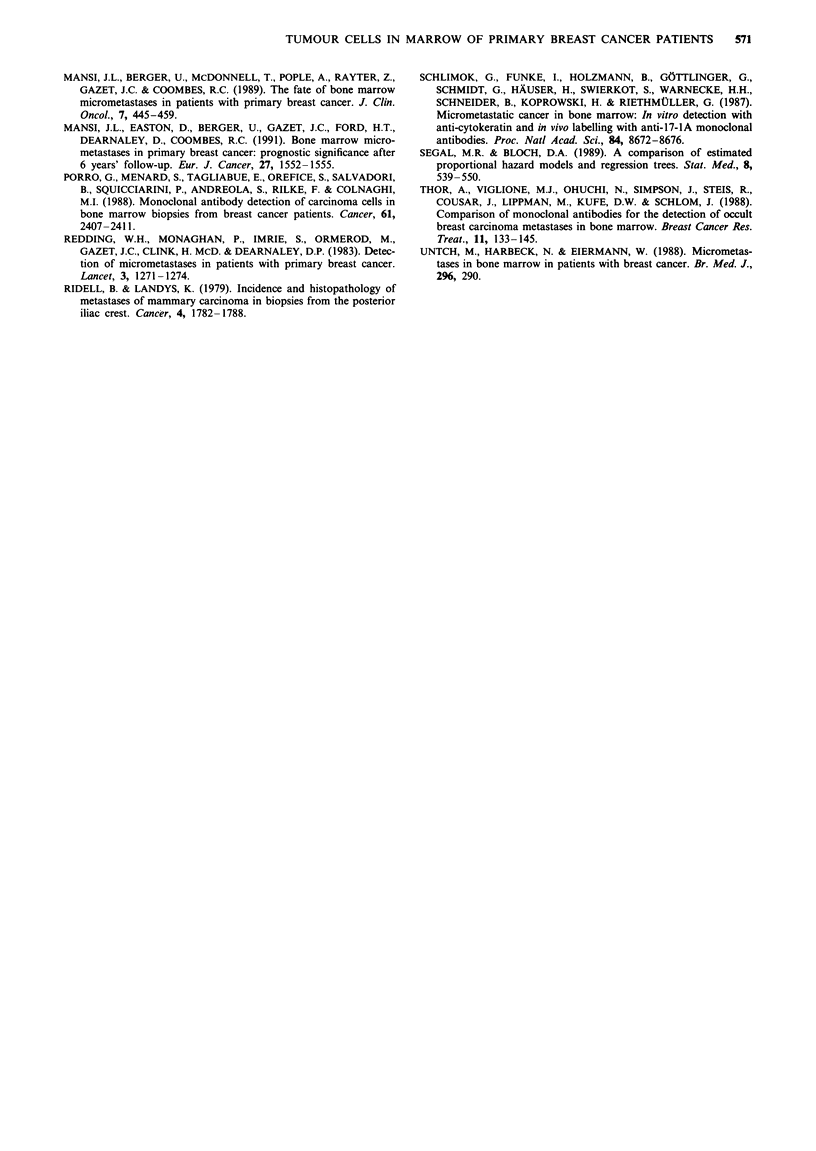# Tumour cell detection in the bone marrow of breast cancer patients at primary therapy: results of a 3-year median follow-up.

**DOI:** 10.1038/bjc.1994.103

**Published:** 1994-03

**Authors:** N. Harbeck, M. Untch, L. Pache, W. Eiermann

**Affiliations:** Department of Obstetrics and Gynaecology, Klinikum Grosshadern, Ludwig-Maximilians-Universität, Munich, Germany.

## Abstract

We examined bone marrow aspirates from 100 metastasis-free primary breast cancer patients. In 38/100 patients (38%), tumour cells were detected in the marrow using an immunocytochemical technique with a cocktail of two monoclonal antibodies: anti-EMA and anti-cytokeratin. Median follow-up was 34 months: 15/38 (39%) tumour cell-positive patients have since relapsed, but only 9/62 (15%) tumour cell-negative patients. The median interval between tumour cell detection and relapse was 11.4 months. No statistically significant correlation existed between tumour cell presence and 'established' prognostic factors. However, relapse-free survival was significantly shorter in tumour cell-positive patients. Multivariate analysis showed tumour cell presence as a strong, significant prognostic factor for relapse-free as well as overall survival. We conclude that screening for tumour cells in bone marrow of primary breast cancer patients identifies high-risk patients for early relapse. In particular, patients with node-negative tumours who have tumour cells in their bone marrow may require subsequent systemic therapy.


Br. J. Cancer (1994), 69, 566-571 © Macmillan Press Ltd., 1994

### Tumour cell detection in the bone marrow of breast cancer patients at primary therapy: results of a 3-year median follow-up

N. Harbeck[1,2], M. Untch[1], L. Pache[2] & W. Eiermann[1]

[1]Department of Obstetrics and Gynaecology, Klinikum Grosshadern, Ludwig-Maximilians-Universität, Munich, Germany; [2]Department of Obstetrics and Gynaecology, Klinikum Rechts der Isar, Technische Universität, Munich, Germany.

**Summary** We examined bone marrow aspirates from 100 metastasis-free primary breast cancer patients. In 38/100 patients (38%), tumour cells were detected in the marrow using an immunocytochemical technique with a cocktail of two monoclonal antibodies: anti-EMA and anti-cytokeratin. Median follow-up was 34 months: 15/38 (39%) tumour cell-positive patients have since relapsed, but only 9/62 (15%) tumour cell-negative patients. The median interval between tumour cell detection and relapse was 11.4 months. No statistically significant correlation existed between tumour cell presence and 'established' prognostic factors. However, relapse-free survival was significantly shorter in tumour cell-positive patients. Multivariate analysis showed tumour cell presence as a strong, significant prognostic factor for relapse-free as well as overall survival. We conclude that screening for tumour cells in bone marrow of primary breast cancer patients identifies high-risk patients for early relapse. In particular, patients with node-negative tumours who have tumour cells in their bone marrow may require subsequent systemic therapy.

Six to nine per cent of white women in Europe and North America will develop breast cancer in their lifetime. Researchers in the USA even talk about a breast cancer epidemic (Greenspan, 1987). Despite all progress made over the last decades in diagnosis and treatment, overall survival has not significantly increased. The median 10-year survival rate is still less than 50% (Henderson & Canellos, 1980). Even among a presumably 'low-risk' population like node-negative patients there is a death rate of about 25% over a decade (McGuire *et al.*, 1989).

At primary therapy less than 10% of all breast cancer patients present with clinically detectable distant tumour spread (Henderson & Canellos, 1980). Therefore, indirect prognostic criteria such as tumour size, lymph node and receptor status, grading or DNA analysis are used to characterise the individual patient's risk for relapse. Based on this risk analysis, adjuvant systemic therapy is administered. Potentially severe short- and long-term side-effects might be associated with adjuvant therapy. However, recent reports demand an even larger group of patients (i.e. node-negative ones) to be treated in an adjuvant setting (Fisher *et al.*, 1989*a*; Ludwig Breast Cancer Study Group, 1989). Better prognostic criteria are therefore urgently needed to identify those patients who will actually benefit from adjuvant therapy. This would allow a more individualised risk-benefit analysis (McGuire *et al.*, 1990).

One approach to such an improved risk analysis is the search for micrometastatic tumour spread at primary therapy. The skeletal system is the predominant relapse location in breast cancer (Coombes *et al.*, 1983). It is therefore quite obvious to search there for early distant spread. Conventional biopsies enabled tumour cell detection in the bone marrow of 3.9% apparently metastasis-free patients (Ridell & Landys, 1979).

This percentage was considerably raised by the use of immunocytochemical methods (Dearnaley *et al.*, 1981). Encouraged by the results of the London group from the Ludwig Institute (Dearnaley *et al.*, 1981; Redding *et al.*, 1983), our group started in 1984 to screen for tumour cells in the bone marrow of primary breast cancer patients. After a median follow-up of almost 3 years, we now seek to evaluate the significance of this tumour cell detection as a new prognostic factor in breast cancer.

Correspondence: N. Harbeck, Frauenklinik der Technischen Universität, Ismaningerstrasse 22, 81675 Munich, Germany.
Received 27 July 1993; and in revised form 5 October 1993.

### Patients and methods

### Patients

From October 1984 until February 1990, 115 primary breast cancer patients between the age of 32 and 77 years (median 54 years) were entered into this study. Before surgery, all patients were screened for distant metastases by clinical examination, blood tests [CEA, CA 15-3, γ-glutylamyltransferase (γ-GT), alkaline phosphatase (AP)], liver ultrasound, chest radiography and bone scan. Fifteen patients with overt metastases (i.e. stage M1) were excluded from further statistical evaluation. Depending on the size of the tumour, all patients underwent either modified radical mastectomy or breast-preserving surgery (with subsequent radiation) as well as axillary lymph node dissection. Bone marrow aspirates were taken immediately after surgery from six sites: upper and lower sternum as well as left and right anterior and posterior iliac crest. Hormone receptor status of the primary tumour was determined either by DCC (dextran charcoal assay) or by immunohistochemistry ('ERICA', Abbot, Chicago, USA).

All patients had their primary treatment as well as their follow-up visits at our hospital. Thus, a consistency of treatment was achieved. Every node-positive patient received either adjuvant chemo- (standard CMF) or adjuvant hormone (tamoxifen) therapy depending on receptor status and menopausal state. After primary therapy, patients had regular check-ups with thorough clinical examination and blood tests (CEA, CA-153, AP, γ-GT) every 3 months, as well as a chest radiography and a liver ultrasound every 6 months, and a bone scan every year.

### Immunocytochemical staining procedure

From each aspiration site 4-6 ml of bone marrow was taken. In the first 16 patients the aspirates of each site were analysed separately. However, no correlation between aspiration site and relapse location could be found. Therefore, we then started to pool the aspirates for further preparation. The bone marrow suspension was separated on a Lymphoprep gradient (density 1.077) as described by Böyum (1968). The interphase layer was separated, washed, resuspended and smeared onto 20-40 glass slides at a final concentration of about $2 \times 10^7$ cells $ml^{-1}$. The slides were then wet fixed in absolute ethanol and stored at $-20°C$. For the staining procedure we modified the technique introduced by Dearnaley *et al.* (1981). We used the following monoclonal primary antibodies rather than polyclonal antibodies for the indirect immunocytochemical staining procedure:

(1) anti-EMA (clone E-29, Dako, Hamburg, Germany);
(2) a cocktail of two monoclonal antibodies, anti-EMA (see above) and anti-cytokeratin (Moll's No. 8, 18, 19, Becton Dickinson, Heidelberg, Germany);
(3) 12-H-12, a biotinylated antibody against the breast cancer associated glycoprotein TAG-12 (kindly provided by Professor Kaul, Heidelberg University, Germany).

After blocking endogenous alkaline phosphatase activity with 20% acetic acid and 2.28% periodic acid, the slides were incubated with the primary antibodies. Subsequently the secondary antibody was applied – a rabbit anti-mouse antibody conjugated to alkaline phosphatase (Dako, Hamburg, Germany). For the 12-H-12 antibody, alkaline phosphatase-labelled avidin was used instead. Between the staining steps, the slides were washed in PBS buffer. As substrate for visualisation we used fast red TR salt (Sigma, Munich, Germany) dissolved in Tris buffer pH 8.2 and naphthol-AS-MX-phosphate (in *N*-*N*-dimethylformamide), adding 2% levamisole to again block endogenous alkaline phosphatase activity. The slides were then counterstained with Mayer's haemalaun and coverslips applied. To facilitate screening of the slides, we evaluated them on a monitor linked to an automatic slide table ('Prodyscope', Will, Wetzlar, Germany). Cells were only classified as tumour cells if they showed immunocytochemical staining as well as the morphological criteria of tumour cells.

### *Statistical methods*

Statistical data evaluation was performed with the EDA ('easy data analysis') software package (Professor Köpcke, IBE, Munich, Germany, 1989). A 95% confidence interval was used for all statistical tests. Thus, only $P$-values $<0.05$ were considered to be statistically significant. The $\chi^2$-method was applied to assess the correlation between tumour cell detection and 'established' prognostic factors. Analysis and plotting of survival data was performed by means of the Kaplan–Meier estimate. The Mantel–Cox test was used for calculation of survival probabilities (Kaplan & Meier, 1958). Optimal cut-off values for the prognostic factor 'tumour size', i.e. for classification of 'small' and 'large' tumours were calculated by the CART method (classification and regression trees) (Segal & Bloch, 1989) and the maximum likelihood method. To compare the predictive value of the prognostic factors for relapse-free as well as overall survival, we performed a multivariate analysis applying 'Cox's proportional hazard model' (Cox, 1972) and the BMDP software package (Dixon, 1981).

### Results

### *Tumour cell detection*

At the time of primary therapy, tumour cells in the bone marrow were detected in 38 of the 100 breast cancer patients (38%) without clinically detectable metastases (i.e. stage M0).

### *Correlation with established prognostic factors*

Table I shows the correlation between tumour cell presence in the bone marrow and 'established' prognostic factors: tumour size, lymph node involvement, menopausal state, receptor status, grading and histological type of the tumour.

**Table I** Correlation between detection of tumour cells in the bone marrow and other prognostic factors (100 patients at stage M0)

| *Prognostic factor* | *Tumour cell positive (n = 38)* | *Tumour cell negative (n = 62)* | *Correlation ($\chi^2$ test)* |
|---|---|---|---|
| Tumour size | | | $P = 0.65$ |
| pT1 ($n = 47$) | 18 (38%) | 29 (62%) | |
| pT2 ($n = 36$) | 12 (33%) | 24 (67%) | |
| pT3 ($n = 6$) | 2 | 4 | |
| pT4 ($n = 8$) | 5 | 3 | |
| pTx ($n = 3$) | 1 | 2 | |
| Lymph node status | | | $P = 0.24$ |
| pN0 ($n = 39$) | 19 (49%) | 20 (51%) | |
| 1–3 lymph nodes ($n = 28$) | 7 (25%) | 21 (75%) | |
| >3 lymph nodes ($n = 32$) | 11 (34%) | 21 (66%) | |
| pNx ($n = 1$) | 1 | 0 | |
| Oestrogen receptor | | | $P = 0.40$ |
| Positive ($n = 56$) | 18 (32%) | 38 (68%) | |
| Negative ($n = 31$) | 14 (45%) | 17 (55%) | |
| Not tested ($n = 13$) | 6 | 7 | |
| Progesterone receptor | | | $P = 0.73$ |
| Positive ($n = 38$) | 15 (39%) | 23 (61%) | |
| Negative ($n = 49$) | 17 (35%) | 32 (65%) | |
| Not tested ($n = 13$) | 6 | 7 | |
| Menopausal state | | | $P = 0.33$ |
| Premenopausal ($n = 51$) | 17 (33%) | 34 (67%) | |
| Post-menopausal ($n = 49$) | 21 (43%) | 28 (57%) | |
| Grading | | | $P = 0.35$ |
| G1 ($n = 2$) | 0 | 2 | |
| G2 ($n = 62$) | 25 (40%) | 37 (60%) | |
| G3 ($n = 27$) | 8 (30%) | 19 (70%) | |
| Gx ($n = 9$) | 5 | 4 | |
| Histological type | | | $P = 0.13$ |
| Ductal carcionoma ($n = 71$) | 22 (31%) | 49 (69%) | |
| Lobular carcinoma ($n = 11$) | 8 | 3 | |
| Inflammatory carcinoma ($n = 3$) | 1 | 2 | |
| Other ($n = 15$) | 7 | 8 | |

Patients under the age of 50 years were classified as premenopausal and patients of 50 years and older as postmenopausal. None of the prognostic factors showed a statistically significant correlation with the presence of tumour cells.

*Correlation with follow-up data*

*Metastasis* Table II shows the correlation between tumour cell presence and patient outcome over a median follow-up period of 34 months (maximum 65 months, minimum 7 months). Of the total of 100 patients, 24 (24%) have already relapsed, 14 (14%) at distant sites. Tumour cell-positive patients were at significantly higher risk ($P = 0.016$) for relapse than tumour cell-negative patients (39% *vs* 15%). The relapse-free survival data of tumour cell-positive and tumour cell-negative patients is plotted in Figure 1. Nine of the 38 (24%) tumour cell-positive patients have since relapsed at distant sites compared with 5/62 (8.1%) tumour cell-negative patients. Up till now, no tumour cell-negative patient has relapsed solely in the skeletal system; only one tumour cell-negative patient has relapsed in bone and visceral sites simultaneously. In comparison, in seven tumour cell-positive patients the first site of relapse was bone, in six of whom relapse occurred solely in the skeletal system (see Table II). This difference is highly significant ($P = 0.008$). Additionally, within the subgroup of oestrogen receptor-positive patients ($n = 56$), tumour cell presence in the bone marrow also had a significant impact on survival: relapse-free ($P = 0.0086$) as well as overall survival ($P = 0.0271$) was significantly shorter in tumour cell-positive, oestrogen receptor-positive patients.

The median interval between tumour cell detection in the bone marrow and metastasis is 11.4 months (maximum 28 months, minimum 1 month). In total, the median relapse-free survival time was 28 months. However, tumour cell-positive patients had a significantly shorter relapse-free survival than tumour cell-negative ones: 19 months *vs* 33 months ($P = 0.0011$) (see Table II).

*Survival* The overall survival data can be seen in Table III. Sixteen of the total of 100 patients (16%) have already died: nine (24%) in the tumor cell-positive group and seven (11%) in the tumour cell-negative group. In total, the median overall survival time was 33 months, 29 months for tumour cell-positive patients and 36 months for tumour cell-negative patients. The impact of tumour cell presence on overall survival has not yet reached statistical significance ($P = 0.0826$). Figure 2 shows the overall survival curves of tumour cell-positive and tumour cell-negative patients.

*Node-negative patients* A total of 39/100 patients (39%) were free of lymph node involvement at the time of primary therapy. In this subgroup of patients, the percentage tumour cell detection was 49%. A significant correlation existed only between histological type of the primary tumour and tumour cell presence and 'established' prognostic factors in N0 patients. Table IV shows the follow-up data of all 39 node-negative patients. The correlation between tumour cell presence and patient follow-up coincides with the findings in the total population of M0 patients (see Tables II and III): tumour cell-positive patients tend to have a worse outcome than tumour cell-negative ones.

*Multivariate analysis*

Only those prognostic factors exhibiting at least borderline significance for relapse-free or overall survival in the

**Figure 1** Tumour cell presence in the bone marrow and relapse-free survival (RFS) in primary breast cancer ($n = 100$).

**Figure 2** Tumour cell presence in the bone marrow and overall survival (OS) in primary breast cancer ($n = 100$).

**Table II** Relapse after tumour cell detection in the bone marrow (100 initially metastasis-free patients)

| | *Tumour cell positive* (n = *38*) | *Tumour cell negative* (n = *62*) |
|---|---|---|
| Relapse (all locations) | | |
| Node-negative patients ($n = 39$) | 5/19 | 1/20 |
| Node-positive patients ($n = 61$) | 10/19 | 8/42 |
| All patients ($n = 100$): 24% | 15/38 (39%) | 9/62 (15%) |
| Site of relapse | | |
| Bone | 6 | 0 |
| Visceral (lung, liver) | 2 | 3 |
| Soft tissue (skin, nodes) | 0 | 1 |
| Mixed (bone + visceral) | 1 | 1 |
| Distant relapse ($n = 14$): 14% | 9 (24%) | 5 (8.1%) |
| Local relapse ($n = 10$): 10% | 6 (16%) | 4 (6.5%) |
| Time[a] between tumour cell detection and relapse | 11.4 months | – |
| Relapse-free survival[a] all patients: 28 months | 19 months | 33 months |

[a]Median period.

**Table III** Overall survival after tumour cell detection in the bone marrow (100 patients, initially at stage M0)

| | *Tumour cell positive* (n = *38*) | *Tumour cell negative* (n = *62*) |
|---|---|---|
| Overall survival[a] | | |
| All patients: 33 months | 29 months | 36 months |
| Deceased | | |
| Total 16 patients (16%) | 9 (24%) | 7 (11%) |

[a]Median period.

univariate Kaplan–Meier test were considered for multivariate analysis. Since there was a highly significant correlation between tumour size and lymph node status ($P = 0.0007$), only the clinically more important of these two factors, i.e. lymph node status, was entered into multivariate analysis.

Absence of tumour cells in the bone marrow was the strongest predictor for relapse-free survival ($P = 0.0005$), even stronger than lymph node status ($P = 0.0367$). The remaining two parameters, grading and oestrogen receptor status, showed no statistical significance for relapse-free survival (see Table Va).

Histopathological grading had the highest significant impact ($P = 0.0027$) on overall survival, closely followed by tumour cell presence ($P = 0.0170$). However, lymph node involvement and oestrogen receptor status were not statistically significant for the prediction of overall survival (see Table Vb).

### Discussion

The percentage of tumour cell-positive patients (38%) in this study is slightly higher than in our earlier publications (Harbeck *et al.*, 1987; Untch *et al.*, 1988) or the percentage reported by some other groups (Mansi *et al.*, 1987; Schlimok *et al.*, 1987; Porro *et al.*, 1988; Ellis *et al.*, 1989; Diel *et al.*, 1990). One explanation might be an improved detection rate by using an antibody cocktail instead of a single monoclonal antibody. Using a similar antibody cocktail, Cote *et al.* (1991) found a similar detection rate of 37%. Unspecific, false-positive staining has been reported with anti-EMA as well as anti-cytokeratin antibodies (Heyderman & McCartney, 1985; Ellis *et al.*, 1989). This underlines the importance of using both immunocytochemical as well as morphological criteria before classifying cells as tumour cells (Berger *et al.*, 1988). There are reports that anti-cytokeratin antibodies are less sensitive in recognising breast cancer metastases than anti-EMA (Thor *et al.*, 1988). Therefore, we used an additional antibody (12-H-12) that does not stain lymphoid cells (Kaul *et al.*, 1989) as a parallel control: cells were only classified as tumour cells if they stained positively with the anti-EMA/anti-cytokeratin cocktail as well as 12-H-12. Another factor contributing to our detection rate might be the use of six aspiration sites (Coombes *et al.*, 1983). Similarly, Diel *et al.* (1990) were able to raise their detection rate from 24% to 32% by increasing the number of aspiration sites from 2 to 6. Based on our earlier observations (confirming the results of Mansi *et al.*, 1987) that the number of detected tumour cells showed no evidence of prognostic impact, we did not take into account the actual number of cells detected. Considering more recent results by Cote *et al.* (1991), it might be advisable for future studies of more patients to include this factor.

The question of whether detected tumour cells are actually micrometastases or whether they are shed by the primary tumour or seeded during surgery without bearing any further clonogenic potential has been raised in the literature (Mansi *et al.*, 1989). In a median interval of 13 months after primary surgery, we repeated marrow aspirates in three initially tumour cell-negative patients: all of them have remained tumour cell-negative, and up until now none of them has relapsed. Porro *et al.* (1988) aspirated bone marrow in 11 of their patients before as well as after primary surgery. None of these patients had a different result after surgery. Thus, they were able to rule out possible tumour cell spread due to surgical manipulation. Yet another argument against a merely artificial spread of tumour cells by surgical intervention is the fairly constant percentage of tumour cell detection in the literature, whether bone marrow aspirates are taken immediately before (Mansi *et al.*, 1987; Porro *et al.*, 1988; Cote *et al.*, 1991) or after (Harbeck *et al.*, 1987; Diel *et al.*, 1992) primary surgery. In our opinion, multiple aspiration sites as well as the use of cocktails of monoclonal antibodies are likely to play a far more important role in an increased detection rate. To examine the viability of the detected tumour cells, we developed a method for establishing cell cultures from the marrow aspirates. So far we have succeeded in growing cell cultures from one tumour cell-positive patient. The identity of the observed clones was proven immunocytochemically. In addition to these laboratory experiments, the follow-up data presented by our group, in accordance with other groups, strongly suggest that a large proportion of these cells may be viable and clonogenic. In our study the relapse rate among tumour cell-positive patients was almost three times that of tumour cell-negative patients.

Our follow-up data over a 5-year period confirm that tumour cell detection in the bone marrow of primary breast cancer patients is a statistically significant prognostic factor for early relapse. Its prognostic value is even higher in predicting bone metastases. Tumour cell-positive patients also seem to have a worse prognosis with regard to overall survival, although this trend is not yet significant. Even within a subgroup defined by 'established' prognostic factors such as oestrogen receptor-positive patients, tumour cell presence characterises high-risk patients for a significantly shorter relapse-free as well as overall survival. In a multivariate setting, tumour cell presence is a statistically

**Table IV** Follow-up data in node-negative patients (39 patients, initially at stage M0)

| | *Tumour cell positive* (n = *19*) | *Tumour cell negative* (n = *20*) |
|---|---|---|
| Relapse (all locations) | 5 | 1 |
| Site of first relapse | | |
| Bone | 2 | 0 |
| Other | 3 | 1 |
| Time between tumour cell detection and metastasis[a] | 9.6 months | – |
| Relapse-free survival[a] | 19 months | 37 months |
| Deceased | 3 | 1 |
| Overall survival[a] | 30 months | 38 months |

[a]Median period.

**Table Va** Relapse-free survival in primary breast cancer ($n$ = 100): multivariate analysis of various prognostic factors

| *Prognostic factor* | *Univariate* P-*value* | *Multivariate* P-*value* | *Relative risk (95% CI)*[a] |
|---|---|---|---|
| Tumour cell presence (yes/no) | 0.0011 | 0.0005 | 4.1 (1.8–9.1) |
| Nodal status (positive/negative) | 0.0448 | 0.0367 | 2.5 (1.0–6.2) |
| ER status (positive/negative) | 0.0609 | 0.2310 | |
| Grading (G1–2/G3) | 0.2585 | 0.2396 | |

[a]Confidence interval.

**Table Vb** Overall survival in primary breast cancer ($n$ = 100): analysis of various prognostic factors

| *Prognostic factor* | *Univariate* P-*value* | *Multivariate* P-*value* | *Relative risk (95% CI)*[a] |
|---|---|---|---|
| Grading (G1–2/G3) | 0.0075 | 0.0027 | 4.7 (1.5–33.4) |
| Tumour cell presence (yes/no) | 0.0826 | 0.0170 | 3.7 (1.3–10.5) |
| Nodal status (positive/negative) | 0.0617 | 0.0730 | 2.8 (0.8–10.2) |
| ER status (positive/negative) | 0.0493 | 0.1404 | |

[a]Confidence interval.

significant prognostic factor for relapse-free as well as overall survival. Its prognostic impact outweighs even that of a clinically important, 'established' prognostic factor such as lymph node status. Cote et al. (1991) also found tumour cell presence to be a better predictor for early recurrence than lymph node status. Mansi et al. (1991) reported after a median follow-up of 76 months that tumour cell detection was a significant prognostic factor for relapse-free as well as overall survival. However, their multivariate analysis showed that this prognostic impact was less than that of tumour size or nodal status.

In contrast to other researchers (Mansi et al., 1992; Diel et al., 1992), we did not obtain a statistically significant correlation between tumour cell detection and 'established' prognostic factors. However, the role of vascular invasion (Mansi et al., 1987) was not evaluated. In our study, keeping in mind that the numbers in some subgroups are still small, tumour cell presence appears to be an independent prognostic factor. Tumour cell-positive patients seem to be fairly evenly distributed among the various risk groups, i.e. no excess of tumour cell-positive patients in a particular risk group was detected. However, there is one somewhat unexpected exception: the percentage of tumour cell-positive patients is even higher among node-negative patients (49%) than among node-positive patients (36%). The follow-up data of the node-negative patients resemble those of the total 100 M0 patients: tumour cell-positive patients tend to have a worse prognosis with shorter relapse-free as well as overall survival. Porro et al. (1988) also reported a higher detection rate in node-negative patients, although the difference in their study is not as evident: 17% vs 14%. Our results tend to confirm their theoretical considerations. By examining bone marrow aspirates, one may discover an alternative route for metastasis that bypasses the axillary lymph nodes via the bloodstream. Thus, there might be two independent but equivalent routes for metastatic spread in breast cancer (Fisher et al., 1989b). This concept, together with the presented follow-up data, emphasises the importance of bone marrow aspirates for identifying an additional risk group of patients – especially among node-negative patients – that might have been missed by sole consideration of the established prognostic factors. Our study also suggests that node-positive, tumour cell-positive patients might benefit from a more intensive form of adjuvant therapy (e.g. high-dose chemotherapy).

Methodological improvements need to be made, since the detection method is still too time-consuming for routine application. Further clinical studies, perhaps on the basis of national or international collaborations, are warranted in order to confirm the international follow-up results presented so far: Only larger patient groups and longer follow-up periods will allow more detailed subgroup analyses, in particular among node-negative patients. Such studies will help to decide whether tumour cell presence is an important prognostic factor by itself or whether it is of more value in a panel of prognostic factors (McGuire et al., 1990). A multicentre study has now been proposed in Germany in which node-negative, tumour cell-positive patients will be randomised to receive adjuvant therapy or not (Funke et al., 1991). The results of this trial will determine whether adjuvant therapy is beneficial in patients with tumour cells in their bone marrow.

We would like to thank Mrs M. Felber, Mrs H. Gottschalk-Deponte and Mrs M. Waldherr for their competent technical support.

### References

BERGER, U., BETTELHEIM, R., MANSI, J.L., EASTON, D., COOMBES, R.C. & NEVILLE, A.M. (1988). The relationship between micrometastases in the bone marrow, histopathologic features of the primary tumor in breast cancer and prognosis. *Am. J. Clin. Pathol.*, **90**, 1-6.

BÖYUM, A. (1968). Separation of leucocytes from blood and bone marrow. *Scand. J. Clin. Lab. Invest.*, **21** (Suppl. 97).

COOMBES, R.C., DEARNALEY, D.P., REDDING, W.H., ORMEROD, M.G., SKILTON, R.A., SLOANE, J.P., IMRIE, S., EDWARDS, A.W., MONAGHAN, P. & NEVILLE, A.M. (1983). Micrometastases in breast cancer. In *Protides of the Biological Fluids*, Vol. 317, Peeters, H. (ed.), pp. 317-323, Pergamon Press: Oxford.

COTE, R.J., ROSEN, P.P., LESSER, M.L., OLD, L.J. & OSBORNE, M.P. (1991). Prediction of early relapse in patients with operable breast cancer by detection of occult bone marrow micrometastases. *J. Clin. Oncol.*, **9**, 1749-1756.

COX, D.R. (1972). Regression models and life tables. *J. R. Stat. Soc.*, **B**, 187-220.

DEARNALEY, D.P., SLOANE, J.P., ORMEROD, M.G., STEELE, K., COOMBES, R.C., CLINK, H. MCD., POWLES, T.J., FORD, H.T., GAZET, J.P. & NEVILLE, A.M. (1981). Increased detection of mammary carcinoma cells in marrow smears using antisera to epithelial membrane antigen. *Br. J. Cancer*, **44**, 85-90.

DIEL, I.J., KAUFMANN, M., COSTA, S.D., KAUL, S., KREMPIEN, B., GOERNER, R. & BASTERT, G. (1990). Prognostische Bedeutung des Tumorzellnachweises im Knochenmark von Patientinnen mit Mammakarzinom. *Geburtsh u Frauenheilk*, **50**, 923-928.

DIEL, I.J., KAUFMANN, M., GOERNER, R., COSTA, S.D., KAUL, S. & BASTERT, G. (1992). Detection of tumor cells in bone marrow of patients with primary breast cancer: a prognostic factor for distant metastasis. *J. Clin. Oncol.*, **10**, 1534-1539.

DIXON, W.J. (ed.) (1981). *BMDP Statistical Software*. pp. 330-334, 555-594. University of California Press: Berkeley.

ELLIS, G., FERGUSON, M., YAMANAKA, E., LIVINGSTON, R.B. & GOWN, A.M. (1989). Monoclonal antibodies for detection of occult carcinoma cells in bone marrow of breast cancer patients. *Cancer*, **63**, 2509-2514.

FISHER, B., CONSTANTINO, J., REDMOND, C., POISSON, R., BOWMAN, D. & others of the NSABP investigation group (1989*a*). A randomized clinical trial evaluating tamoxifen in the treatment of patients with node-negative breast cancer who have estrogen-receptor-positive tumors. *N. Engl. J. Med.*, **320**, 479-484.

FISHER, B., REDMOND, C., FISHER, E.R. & participating NSABP investigators (1989*b*). The contribution of recent NSABP clinical trials of primary breast cancer therapy to an understanding of tumor biology – an overview of findings. *Cancer*, **46**, 1009-1025.

FUNKE, I., FRIES, S. & JAUCH, K.W. (1991). Tumorzellnachweis im Knochenmark: Entscheidungshilfe zur adjuvanten Therapie bei nodal-negativen Patientinnen mit Mammakarzinom. *Chirurg*, **62**, 805-809.

GREENSPAN, E.M. (1987). *The Breast Cancer Epidemic in the United States*. The Chemotherapy Foundation: New York.

HARBECK, N., UNTCH, M. & EIERMANN, W. (1987). Immunocytochemical detection of tumor cells in the bone marrow of breast cancer patients at the time of primary therapy. *Br. J. Cancer*, **56**, 529.

HENDERSON, I.C. & CANELLOS, G.P. (1980). Cancer of the breast. *N. Engl. J. Med.*, **302**, 17-30.

HEYDERMAN, E. & MCCARTNEY, J.C. (1985). Epithelial membrane antigen and lymphoid cells. *Lancet*, **i**, 109.

KAUL, S., WINDECKER, S. & BASTERT, G. (1989). TAG12: Reinigung und Produktion von monoklonalen Zweitantikörpern. *Akt Onk.*, **50**, 329-348.

KAPLAN, E.L. & MEIER, P. (1958). Nonparametric estimation from incomplete observations. *J. Am. Stat. Assoc.*, **53**, 457-481.

LUDWIG BREAST CANCER STUDY GROUP (1989). Prolonged disease-free survival after one course of perioperative adjuvant chemotherapy for node-negative patients. *N. Engl. J. Med.*, **320**, 491-496.

MCGUIRE, W., ABELOFF, M.D., FISHER, B., GLICK, J.H., HENDERSON, I.C. & OSBORNE, C.K. (1989). Adjuvant therapy in node-negative breast cancer. *Breast Cancer Res. Treat.*, **13**, 97-115.

MCGUIRE, W.L., TANDON, A.K., ALLRED, D.C., CHAMNESS, G.C. & CLARK, G.M. (1990). How to use prognostic factors in axillary node-negative breast cancer patients. *J. Natl Cancer Inst.*, **82**, 1006-1015.

MANSI, J.L., BERGER, U., EASTON, D., MCDONNEL, T., REDDING, W.H., GAZET, J.C., MCKINNA, A., POWLES, T.J. & COOMBES, R.C. (1987). Micrometastases in bone marrow in patients with primary breast cancer: evaluation as an early predictor of bone metastases. *Br. Med. J.*, **295**, 1093-1096.

MANSI, J.L., BERGER, U., MCDONNELL, T., POPLE, A., RAYTER, Z., GAZET, J.C. & COOMBES, R.C. (1989). The fate of bone marrow micrometastases in patients with primary breast cancer. *J. Clin. Oncol.*, **7**, 445–459.

MANSI, J.L., EASTON, D., BERGER, U., GAZET, J.C., FORD, H.T., DEARNALEY, D., COOMBES, R.C. (1991). Bone marrow micrometastases in primary breast cancer: prognostic significance after 6 years' follow-up. *Eur. J. Cancer*, **27**, 1552–1555.

PORRO, G., MENARD, S., TAGLIABUE, E., OREFICE, S., SALVADORI, B., SQUICCIARINI, P., ANDREOLA, S., RILKE, F. & COLNAGHI, M.I. (1988). Monoclonal antibody detection of carcinoma cells in bone marrow biopsies from breast cancer patients. *Cancer*, **61**, 2407–2411.

REDDING, W.H., MONAGHAN, P., IMRIE, S., ORMEROD, M., GAZET, J.C., CLINK, H. MCD. & DEARNALEY, D.P. (1983). Detection of micrometastases in patients with primary breast cancer. *Lancet*, **3**, 1271–1274.

RIDELL, B. & LANDYS, K. (1979). Incidence and histopathology of metastases of mammary carcinoma in biopsies from the posterior iliac crest. *Cancer*, **4**, 1782–1788.

SCHLIMOK, G., FUNKE, I., HOLZMANN, B., GÖTTLINGER, G., SCHMIDT, G., HÄUSER, H., SWIERKOT, S., WARNECKE, H.H., SCHNEIDER, B., KOPROWSKI, H. & RIETHMÜLLER, G. (1987). Micrometastatic cancer in bone marrow: *In vitro* detection with anti-cytokeratin and *in vivo* labelling with anti-17-1A monoclonal antibodies. *Proc. Natl Acad. Sci.*, **84**, 8672–8676.

SEGAL, M.R. & BLOCH, D.A. (1989). A comparison of estimated proportional hazard models and regression trees. *Stat. Med.*, **8**, 539–550.

THOR, A., VIGLIONE, M.J., OHUCHI, N., SIMPSON, J., STEIS, R., COUSAR, J., LIPPMAN, M., KUFE, D.W. & SCHLOM, J. (1988). Comparison of monoclonal antibodies for the detection of occult breast carcinoma metastases in bone marrow. *Breast Cancer Res. Treat.*, **11**, 133–145.

UNTCH, M., HARBECK, N. & EIERMANN, W. (1988). Micrometastases in bone marrow in patients with breast cancer. *Br. Med. J.*, **296**, 290.